# Hemoglobin life-threatening value (1.9 g/dl) in good general condition: a pediatric case-report

**DOI:** 10.1186/s13052-021-01146-w

**Published:** 2021-10-07

**Authors:** Emilia Parodi, Lorenzo Riboldi, Ugo Ramenghi

**Affiliations:** 1 Regina Margherita Children’s Hospital, A.O.U. Città della Salute e della Scienza, Torino, Italy; 2 Pediatric Department University of Torino, Piazza Polonia 94, 10126 Torino, Italy; 3 Pediatric and Neonatology Unit, A.O. Ordine Mauriziano, Torino, Italy; 4 Postgraduate School of Pediatrics, University of Torino, Torino, Italy

**Keywords:** Severe anemia, Iron deficiency anemia, Sickle cell anemia, Children, Case report

## Abstract

**Background:**

We report a pediatric patient presenting in good general condition despite a hemoglobin value of 1,9 g/dL, which is normally regarded as life-threatening.

**Case presentation:**

An African 5 years-old girl presented to our Emergency Department (ED) for worsening asthenia, within a clinical picture of good general condition. The hemoglobin value at admission was 1,9 g/dL. The subsequent diagnostic-therapeutic pathway highlighted the presence of two different causes, both well known to be responsible for chronic anemia (with slow reduction of hemoglobin values): iron deficiency anemia (IDA) due to a very low dietary intake of iron-rich foods, and homozygous sickle cell disease (HbSS). She received transfusions of packed red blood cells (overall 15 ml/kg) and subsequently intravenous iron preparations (total amount 200 mg) followed by oral iron supplements. The Hb value at discharge, 10 days after the admission, was 9.8 g/dL.

**Conclusions:**

When approaching a picture of severe anemia, we suggest pediatricians take into consideration clinical conditions rather than laboratory values and to take advantage of detailed anamnestic data in order to make the diagnosis.

## Introduction

Anemia is a serious global public health problem that particularly affects young children; the World Health Organization (WHO) estimates that worldwide, 42% of children less than 5 years of age are anemic. According to the WHO, severe anemia is defined with the cut-off of 7 g/dL in children < 5 years and 8 g/dL in children > 5 years; in the clinical practice, however, the definition of severity of anemia is mainly based on clinical presentation. Due to compensatory mechanisms, in a chronic and slow establishment of anemia, a wide discrepancy between the laboratory value and the presenting clinical condition may be observed.

Herein we describe a child in which the presence of two different causes of chronic anemization (i.e. iron deficiency anemia and homozygous sickle cell disease) led to a very slow decrease of hemoglobin values; the final result of this process was an extremely low and potentially life-threatening hemoglobin value, opposed to satisfactory general condition of the patient.

## Case presentation

A previously healthy 5-years-old girl of African origin presented to the emergency department (ED) in good clinical condition with a 4-day history of low-grade fever as well as progressive and slowly worsening asthenia.

Her medical history showed no previous hospitalization or blood testing. The baby was born in Togo by caesarean section due to fetal distress at 37 weeks of gestational age with a birth weight of 2500 g. Maternal anemia was reported during pregnancy. Four months later the family moved to Italy. The patient was regularly vaccinated and the last trip to Africa dated back to 2 years before the onset of symptoms.

On clinical examination at admission, skin and mucous membranes were markedly pale, such as palms and soles. Good neurological assessment and normal responsiveness and reactivity were observed. Vital parameters were within normal range (HR 110 bpm, RR 20/ min, blood pressure 128/61 mmHg, percutaneous oxygen saturation 100%); capillary refill time was 2 s. No rashes or hemorrhagic manifestations were detected. Cardiac tones were rhythmic with cardiac murmur about 2/6, and shocking wrists.

In contrast to good clinical condition, blood test unexpectedly showed a potentially life-threatening hemoglobin level (Hb 1.9 g/dL).

The baby was diagnosed with severe microcytic anemia (mean corpuscular volume-MCV 64 fl; hematocrit- Htc 8.4%, Red blood cells-RBC 1.760.000/mmc); platelets (PLTs 350.000/mmc) were normal; increased white blood cells (WBC) count and absolute reticulocyte count (ARC) were detected (24,470/mmc and 210.000/mmc, respectively), as usually observed in severe anemia. Peripheral blood smear showed a marked anisopoikilocytosis.

The patient received transfusions of packed red blood cells (overall 15 ml/kg) with a post transfusion Hb level 7.3 g/dL.

Patient’s origins and the clinical picture of severe anemia immediately raised the suspicion of a hidden hemoglobinopathy. Hb High Performance Liquid Chromatography (HPLC) revealed HbS 89.5%. Molecular analysis confirmed the presence of a c.20A > T mutation in homozygous pattern (HbSS).

Pretransfusion laboratory tests were suggestive for severe iron deficiency too (iron binding capacity 6%; Reticulocyte Hemoglobin Content -CHr 15.2 pg).

A careful study of patient’s food history was carried out: the baby had been breastfed until the age of 15 months; subsequently, she had a very selective diet with total rejection of fruit and vegetables and a low meat intake.

The auxological evaluation showed a faltering growth with weight of 14,5 Kg below the 3th centile according to age and sex (z-score − 1.91), height 105.7 cm (25 ° cent, z-score - 0.66) and BMI 13 (< 3 ° cent, z-score - 2.3). Celiac disease was ruled out, thyroid profile and IgG, A and M assay were in the range of normality. Low prealbumin (14 mg/dL) and Vitamin D (7.4 mcg/dL) levels confirmed a malnutritional state and hyperprolactinemia (35.2 ng/ml) was correlated to severe anemia.

Intravenous iron preparations (for a total amount of 200 mg) followed by oral iron supplements were administered with early response demonstrated by the increase in CHr and the progressive normalization of Hb levels.

The baby was discharged 10 days after the admission, in good general condition and with a hemoglobin level of 9.8 g/dl.

**Fig. 1:**
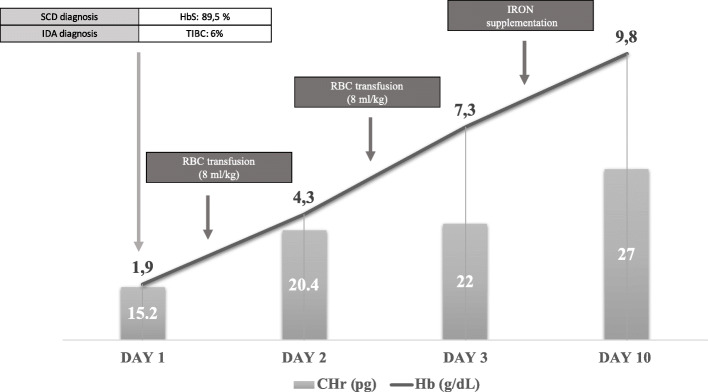
Timeline of diagnostic and therapeutic workout of the episode

The timeline of diagnostic and therapeutic workout of the episode is summarized in Fig. 1.

## Discussion and conclusions

Anemia is a serious global public health problem that particularly affects young children in which the red blood cells’ oxygen-carrying capacity is insufficient to meet the body’s physiological needs. The World Health Organization (WHO) estimates that 42% of children worldwide, less than 5 years of age, are anemic [1, 2].

Anemia is defined as a reduction in hemoglobin concentration or hematocrit below 2 standard deviations (i.e. below 2.5 percentile) per age, race and sex [3]. It represents a common cause of pediatric and hematological consults in children [4]. It is often asymptomatic or paucisymptomatic; thus, most times it is an occasional laboratory finding [5].

According to WHO, severity of anemia is defined depending on Hb value (i.e. < 7 g/dL in children < 5 years and < 8 g/dL in children > 5 years). In clinical practice, however, pediatricians define anemia as severe when a marked reduction in hemoglobin value reflects on clinical condition and onset of symptoms [1].

The timing of development of anemia affects, in turn, the intensity/degree of clinical manifestation. When the decrease of hemoglobin levels is chronic and slow, for example in nutritional deficiencies, patients better tolerate anemia and a wide discrepancy between the laboratory value and the presenting clinical condition may be observed.

Nutritional deficiencies (i.e. iron, folate and vitamins B12) represent one of the most common causes of pediatric anemia. Iron deficiency is the most prevalent nutritional deficiency worldwide [6].

Iron-deficiency anemia (IDA) is the most frequent hematological disease in infancy and childhood. According to World Health Organization statistics, 43% of children worldwide (273 million preschool-age children) are iron deficient; in industrialized countries, 17% of children under 5 years of age suffer from IDA [1]. Factors leading to IDA in children include inadequate dietary iron intake, intestinal malabsorption, and blood losses [7].

In the context of inadequate iron intake, a focused dietary history is an important screening tool, more accurate than an isolated measurement of hemoglobin level, as recently reported in an italian prospective, multicenter study [8]. In a study among 305 healthy African-American children aged one to five years a brief dietary history revealed a 97% negative predictive value for IDA [9].

In our patient, a detailed nutritional evaluation including food history, auxological examination and nutritional indices assessment (i.e. total iron binding capacity, prealbumin and vitamin D levels) confirmed a severe picture of faltering growth.

Pediatric cohorts with severe IDA and extremely low hemoglobin levels have been described in literature so far [10].

However, to the best of our knowledge, for the first time we report an occasional and unexpected diagnosis of IDA in which the child reached potentially life-threatening Hb values (Hb 1.9 g/dl) without important signs or symptoms, suggesting another concomitant pathogenetic mechanism.

In our patient, the ethnicity and the clinical picture of anemia raised immediately the suspicion of a hidden hemoglobinopathy.

The association between nutritional deficiency and Sickle cell disease (SCD) leading to severe anemia has already been reported in African hospitalized children in Nigeria and Tanzania [11, 12].

SCD is the most important hemoglobinopathy worldwide in terms of frequency (400.000 infants are born each year with SCD) and social impact (SCD il responsible for 5–16% of mortality in children younger than 5 years) [13].

The disease is endemic in several Africans countries (most frequently sub-Saharan Africa) and in some parts of Sicily, Greece, southern Turkey, and India. It has recently been recognized as a global public health problem by the World Health Organization, as the current phenomenon of immigration has contributed to a worldwide diffusion of the disease.

SCD is a chronic hemolytic anemia caused by a mutation in β-globin subunit of hemoglobin. Clinical manifestations of SCD include symptoms related to anemia, repeated infections and periodic episodes of pain (VOC, vaso-occlusive crises) [14]. However, both the phenotype of the disease and the onset of symptoms are heterogeneous and may have a pronounced variability among patients. In geographical areas in which a neonatal national screening program for SCD has not been established (i.e. our region in Northern Italy), pediatricians are warranted to have a high suspicion of the disease and screening for HbS should be considered in all patients originating from endemic countries [15].

However, the observed extreme low levels of hemoglobin would hardly have been explained by SCD alone. Compensatory increase in red blood cell production and adaptation to a lower hemoglobin level (approximately 8 to 10 g/dL) are usually sufficient to prevent major symptoms of anemia in most patients. The concomitant presence of other contributing factor, such as folate or iron deficiency, can determine the breakdown of such unstable balance [16], as observed in our patient.

In conclusion, we report an emblematic example that underline how the slower the anemia is established, the more unbelievable hemoglobin levels are asymptomatically reached.

In our patient, the diagnosis of severe anemia might be considered an occasional laboratory finding as the child unexpectedly reached potentially life-threatening Hb values (Hb 1.9 g/dl) without important signs or symptoms (except for asthenia).

This extreme presentation was the consequence of a very slow decrease of hemoglobin values occurring over years, due to the simultaneous presence of two different well known causes of chronic anemization during childhood: iron deficiency anemia and homozygous sickle cell disease.

When approaching a picture of severe anemia, we suggest pediatricians to take into consideration clinical conditions rather than laboratory values and to take advantage of detailed anamnestic data in order to make the diagnosis.

## Data Availability

Not applicable.

## Abbreviations

ED: Emergency department
IDA: iron deficiency anemia
WHO: World Health Organization
SCD: Sickle cell disease
